# Inhibition of phosphotidylinositol-3 kinase pathway by a novel naphthol derivative of betulinic acid induces cell cycle arrest and apoptosis in cancer cells of different origin

**DOI:** 10.1038/cddis.2014.387

**Published:** 2014-10-09

**Authors:** R Majeed, A Hamid, P L Sangwan, P K Chinthakindi, S Koul, S Rayees, G Singh, D M Mondhe, M J Mintoo, S K Singh, S K Rath, A K Saxena

**Affiliations:** 1 Cancer Pharmacology Division, CSIR-Indian Institute of Integrative Medicine, Jammu, India; 2 Bio-organic Chemistry Division, CSIR-Indian Institute of Integrative Medicine, Jammu, India; 3 PK-PD Division, CSIR-Indian Institute of Integrative Medicine, Jammu, India

## Abstract

Betulinic acid (BA) is a pentacyclic triterpenoid natural product reported to inhibit cell growth in a variety of cancers. However, the further clinical development of BA got hampered because of poor solubility and pharmacological properties. Interestingly, this molecule offer several hotspots for structural modifications in order to address its associated issues. In our endeavor, we selected C-3 position for the desirable chemical modification in order to improve its cytotoxic and pharmacological potential and prepared a library of different triazoline derivatives of BA. Among them, we previously reported the identification of a potential molecule, that is, *3{1*N*(5-hydroxy-naphth-1yl)-1*H*-1,2,3-triazol-4yl}methyloxy betulinic acid* (HBA) with significant inhibition of cancer cell growth and their properties. In the present study, we have shown for the first time that HBA decreased the expression of phosphotidylinositol-3 kinase (PI3K) p110*α* and p85*α* and caused significant downregulation of pAKT and of NF*κ*B using human leukemia and breast cancer cells as *in vitro* models. Further it was revealed that PI3K inhibition by HBA induced cell cycle arrest via effects on different cell cycle regulatory proteins that include CDKis cyclins and pGSK3*β*. Also, this target-specific inhibition was associated with mitochondrial apoptosis as was reflected by the increased expression of mitochondrial bax, downregulated bcl2 and decreased mitochondrial levels of cytochrome *c*, together with reactive oxygen species generation and decline in mitochondrial membrane potential. The apoptotic effectors such as caspase 8, caspase 9 and caspase 3 were found to be upregulated besides DNA repair-associated enzyme, that is, PARP cleavage caused cancer cell death. Pharmacodynamic evaluation revealed that both HBA and BA were safe upto the dose of 2000 mg/kg body weight and with acceptable pharmacodynamic parameters. The *in vitro* data corroborated with *in vivo* anticancer activity wherein Ehrlich solid tumor showed that HBA as a more potent agent than BA without any body weight loss and mortality.

Natural products isolated from plant sources have been used extensively in traditional medicine for the treatment of innumerable diseases.^1,2^ Within the sphere of cancer, a number of important new commercialized drugs have been obtained from natural sources either by structural modification of natural compounds or by the synthesis of new compounds using natural compound as model.^3^ The huge structural diversity of natural compounds and their bioactivity potential have meant that several products isolated from plants can serve as lead compounds for improvement of their therapeutic potential by molecular modification.^4^ Betulinic acid (BA) is a naturally occurring triterpenoid with a potential of inducing apoptosis in a variety of malignancies and show remarkable selectivity for tumor cells over non-transformed cells.^5^ The proapoptotic effects of BA have been characterized by several markers of apoptosis, including cleavage of various caspases and the nuclear protein poly-ADP ribose polymerase (PARP).^6^ BA has been reported to induce cancer death via induction of mitochondrial apoptotic pathway.^7^ It shows inhibition of protein specificity transcription factors, activates the stress kinases p38, acts as potent inhibitor of mammalian type 1 DNA topoisomerase, inhibits transcription factor nuclear factor kappa B (NF*κ*B) and shows apoptosis in a p53- and CD95-independent manner.^8, 9, 10^ In addition, triterpenoids are reported to induce cell cycle arrest and apoptosis via the modulation of phosphotidylinositol-3 kinase (PI3K)/AKT pathway, for example, Tanshinone IIA, (Tan IIA) reduced the expression PI3K p85 subunit, and the phosphorylation of AKT and mammalian target of rapamycin in a concentration-dependent manner. The PI3K signaling pathway has established the central role in several cellular processes critical for cancer cell proliferation, including growth, survival, motility, cell cycle progression and metabolism.^11^ Moreover, there are reports that demonstrate the modulation of bcl-2, bax, cyclin D1 and pGSK3*β* expression after BA treatment in several cancer cell lines.^12^ GSK3*β* is a substrate of the PI3K/AKT pathway that is constitutively active in unstimulated cells, and AKT is the kinase primarily responsible for phosphorylation of GSK3*β* at Ser.^9^ There is growing evidences which support the notion that the activation of PI3K/AKT is associated with different events of leukemia and breast cancer and also that class I PI3Ks are highly expressed in breast cancer cell lines and particularly p110*α* representing PI3K*α* enzyme.^13,14^ However, no report has been found suggesting inhibition of PI3K/AKT pathway by BA or any of its structural analogs till date. In spite of tremendous biological activities, further clinical development of BA is greatly hampered because of its poor solubility and poor pharmacokinetic properties.^15^ BA has also been reported to show weak metabolic stability with >60% of the compound getting metabolized leading to low plasma concentrations. The poor permeability coupled with poor aqueous solubility suggests that these compounds may be unsuitable for oral administration.^16^ Therefore much work has been focused on modification of BA on the C-3 and/or C-28 positions in order to increase its hydro solubility and thereby possibly biological properties.^17,18^ In this context, C-3-modified BA derivatives proved to have better *in vivo* anti-tumor efficacy as compared with BA.^19^

With our success toward the synthesis of a library of semi-synthetic analogs of BA to achieve better efficacy and lesser toxicity, chemical modification was done by targeting position 3 of ring A (Figure 1a), one of the hot spots of the molecule which could lead to the generation of a triazole derivative (*3{1*N*(5-hydroxy-naphth-1yl)-1*H*-1,2,3-triazol-4yl}methyloxy betulinic acid* (HBA)) of BA, the potent anticancer candidate with significant apoptotic effects.^20^ Triazole compounds has attracted considerable attention for the past few decades due to their chemotherapeutical value^21^ due to which the anticancer potential of HBA became obvious. However, the molecular mechanism behind its functioning is still not clear. In this communication, we have tried to evaluate the mechanistic role of HBA, a hydroxyl derivative of BA, on apoptosis and cell cycle under *in vitro* conditions and also to evaluate the pharmacodynamics profile besides tumor regression potential of HBA in comparison to BA under *in vivo* conditions.

## Results

### Cytotoxicity profile of BA and its derivatives

**Figure 1:**
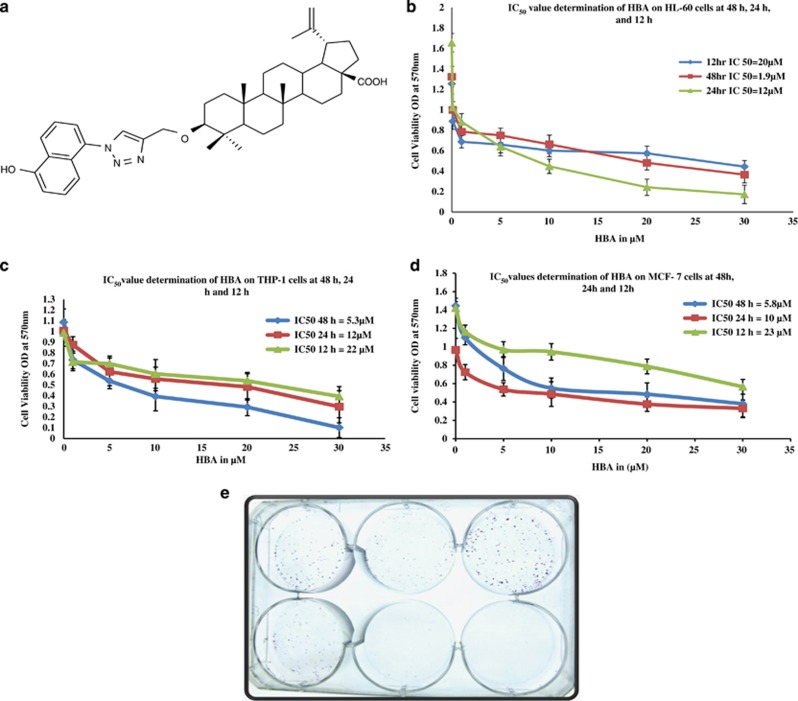
(**a**–**e**) Effect of HBA on proliferation of human leukemia cell lines (HL-60 and THP-1) and breast cancer cell line MCF-7. HL-60, THP-1 and MCF-7 cells (15 × 10^3^) were seeded in 96-well plate. HBA and CBA were added to the cells at different concentrations, whereas the untreated control received the vehicle only. MTT dye was added and OD was measured as described in the Materials and Methods. Data are Mean±S.D. (*n*=6 wells) and representative of two similar experiments. (**e**) *In vitro* clonogenic assay performed on MCF-7 cells. Cells were seeded in six-well plates. After 24 h, the cells were treated with the indicated concentrations of HBA for 24 h . After the completion of the treatment, the plates were placed in an incubator for a time equivalent to at least six potential cell divisions (to give colonies of >50 cells). After fixation, the cells were stained with 0.5% crystal violet for 30 min and allowed to dry at room temperature

BA and its different structural analogs were screened against different human cancer cell lines to evaluate their cytotoxic effect. During preliminary screening at 50 *μ*M concentration, most of the cancer cell lines were found to be sensitive to BA and its derivatives, with many of them exhibiting ≥90 inhibition against various cancer cell lines. At 10 *μ*M concentration, BA was not found to be toxic against any of the cancer cell line. However, many of its derivatives produced concentration-dependent growth inhibition on several cancer cell lines. Leukemia cells (HL-60 and THP-1) and breast cancer cells (MCF-7) proved most sensitive toward the semi-synthetic analogs, in particular HBA showing highly significant cytotoxic effect.^20^ The antiproliferative effects of HBA was further investigated in HL-60, THP-1 and MCF-7 cells in order to get the concentration- and time-dependent IC_50_ values. Treatment of all the three cell lines with HBA produced concentration- and time-dependent inhibition of cell proliferation, with IC_50_ values of approximately 1.9, 12 and 23, 5.3, 12, 22 and 5.8, 10 and 23 *μ*M at 48, 24 and 12 h on HL-60, THP-1 and MCF-7, respectively (Figures 1b–d). HBA has been found to inhibit colony formation in THP-1 cells as reported previously by us. In the present case, we have found that MCF-7 cells have also been restricted for their colony-formation capability at 10 and 20 *μ*M concentrations.

### HBA-mediated downregulation in the expression of PI3K p110*α*, p85*α* and pAKT in HL-60, THP-1 and MCF-7 cells

**Figure 2:**
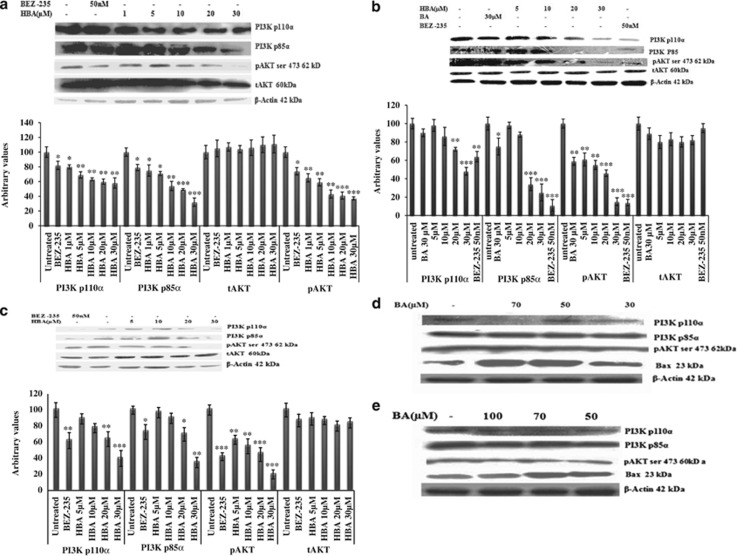
(**a**–**e**) Immunoblot analysis for the expression PI3K p110*α* and p85 in HBA-treated HL-60, THP-1 and MCF-7 cells. Whole-cell lysate was prepared, and protein (100 *μ*g) was resolved on 12% SDS-PAGE gel for western blottig analysis as described in Materials and Methods. Cells (2 × 10^6^) were treated with the indicated concentrations of HBA and BA for 24 h time periods and analyzed by using specific antibodies. Equal protein loading was confirmed by striping and reprobing the same PVDF membrane with antibody against *β*-actin protein. Data are representative of one of two similar experiments. ***P<0.001,**P<0.01, *P<0.05 *versus* control using Student's *t*-test

The PI3K signaling pathway has the central role in several cellular processes critical for cancer cell proliferation, including metabolism, growth, survival, motility and cell cycle progression.^22^ Keeping this in view, we evaluated the expression of p110*α* and p85 isoforms of PI3K in HL-60, THP-1 and MCF-7 cells. We found significant downregulation of both p110*α* and p85*α* isoforms of PI3K even at low micromolar concentration of HBA in all the three cancer cell lines (Figures 2a–c). Further, significant reduction in the phosphorylated AKT (pAKT) protein expression has been observed in all the three cancer cell lines, which is the most immediate substrate of PI3K. However, we could not address any significant changes in the total AKT (tAKT) levels. BA caused significant decrease in the expression of PI3K p110*α*, p85 and pAKT but at very higher concentration, that is, 50, 70 and 100 *μ*M in HL-60 and MCF-7 cells (Figures 2d and e). At 30 *μ*M, BA could not downregulate any of the three proteins; however, at this concentration HBA has downregulated all the three proteins significantly. BEZ-235 a well-known dual PI3K/AKT inhibitor,^23^ was used as a positive control to validate the results that produced significant PI3K p110*α* and p85*α* and pAKT inhibition when used at 50 nM concentration.

### HBA-induced downregulation in the expression of cell cycle regulators cyclins D, E and A

**Figure 3:**
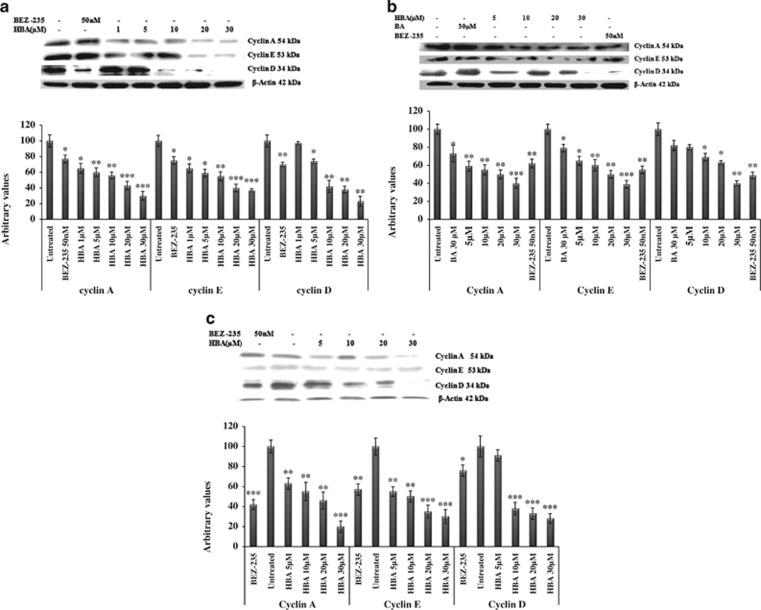
(**a**–**c**) Influence of HBA on the expression of proteins involved in cell cycle regulation. HL-60, THP-1 and MCF-7 cells were treated with the indicated concentrations of HBA for 24 h. *β*-Actin was used as an internal control to represent the same amount of proteins applied for SDS-PAGE. Specific antibodies were used for the detection of cyclins A, D and E levels. Data are representative of one of two similar experiments. ***P<0.001, **P<0.01, *P<0.05 *versus* control using Student's *t*-test

Deregulated cell cycle control is a fundamental aspect of cancer;^24^ therefore we were intended to look into the expression level of the different cyclins. Expression studies done using HL-60, THP-1 and MCF-7 cells revealed downregulation in the expression of cyclins D, E and A in a concentration-dependent manner (Figures 3a–c). In the HL-60 and THP-1 cells compared with the untreated control, cyclin D protein levels were significantly decreased (Figures 3a and b). In MCF-7 cells, the cyclin D expression got significantly decreased even at lower concentration (Figure 3c). In THP-1 cells, the parent BA molecule could not downregulate cyclin D even at 30 *μ*M concentration (Figure 3b). The cyclin E protein is an important cell cycle regulator and is primarily detected during the late G1 phase of the cell cycle. In the present study, we have found significant decrease in the expression of cyclin E protein in HL-60, THP-1 and MCF-7 cells treated with the indicated concentrations of HBA (Figures 3a–c). BA also caused moderate changes in the expression of cyclin E protein at 30 *μ*M concentration (Figure 3b). The overexpression of cyclin A has been linked to the development and progression of majority of cancers.^25^ In HL-60 cells, treatment with HBA caused significant decrease in the expression of cyclin A protein (Figure 3a). Also in THP-1 cells highly significant decrease in the expression of cyclin A has been observed. However, BA when compared with the untreated control caused marginal change in the expression of cyclin A (Figure 3b). In MCF-7 breast cancer cell line, treatment of cells with HBA caused significant inhibition in the expression of cyclin A protein when compared with the untreated MCF-7 cells (Figure 3c). Thus, in the present study, HBA decreased the levels of cyclins D, E and A in a concentration-dependent manner, indicating that treatment with HBA leads to an inhibition of PI3K activity, followed by reduced activation of AKT, which then influences downstream cell cycle regulators that are directly or indirectly regulated by activated AKT.

### Upregulation of p21 and p27 and inhibition of pGSK3*β*-induced cell cycle arrest in HL-60, THP-1 and MCF-7 cells

**Figure 4:**
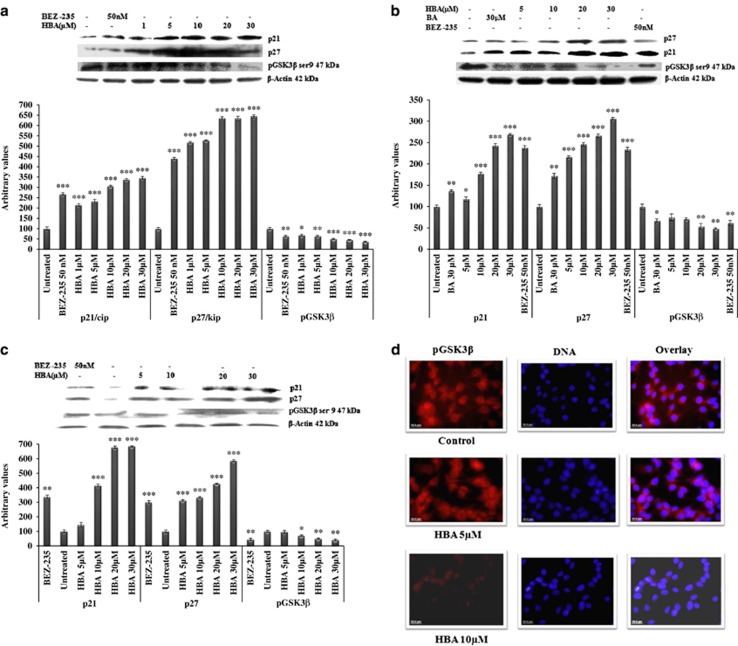

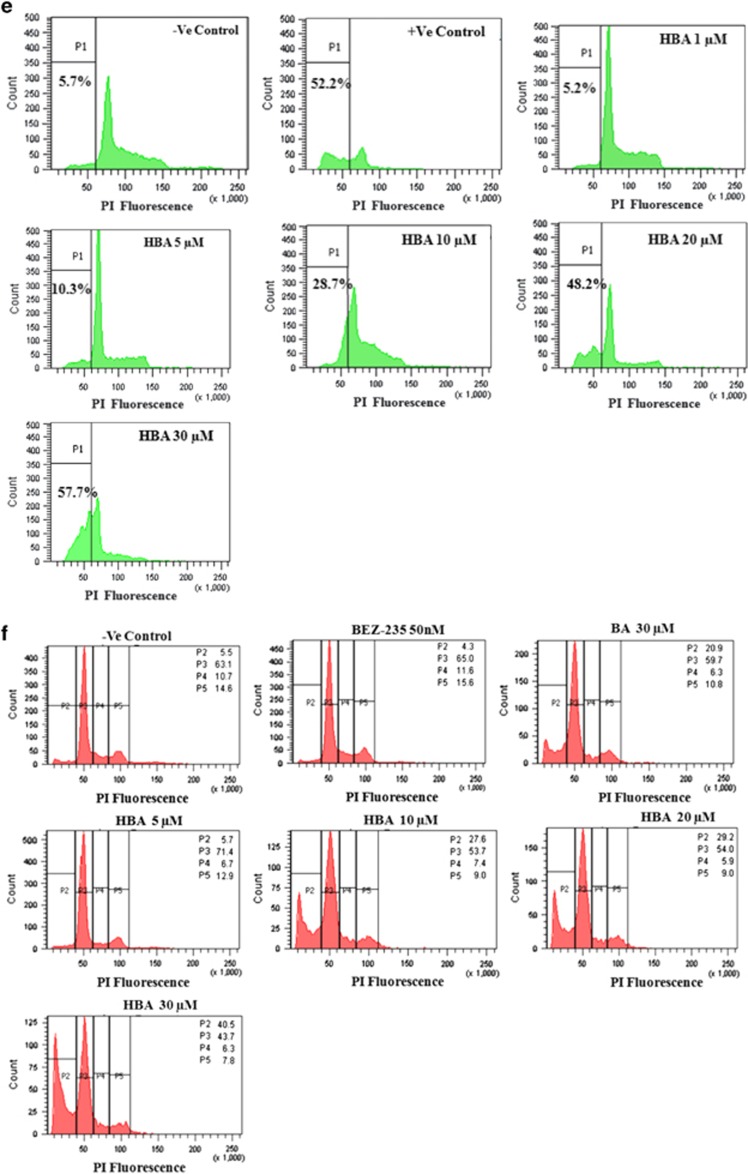

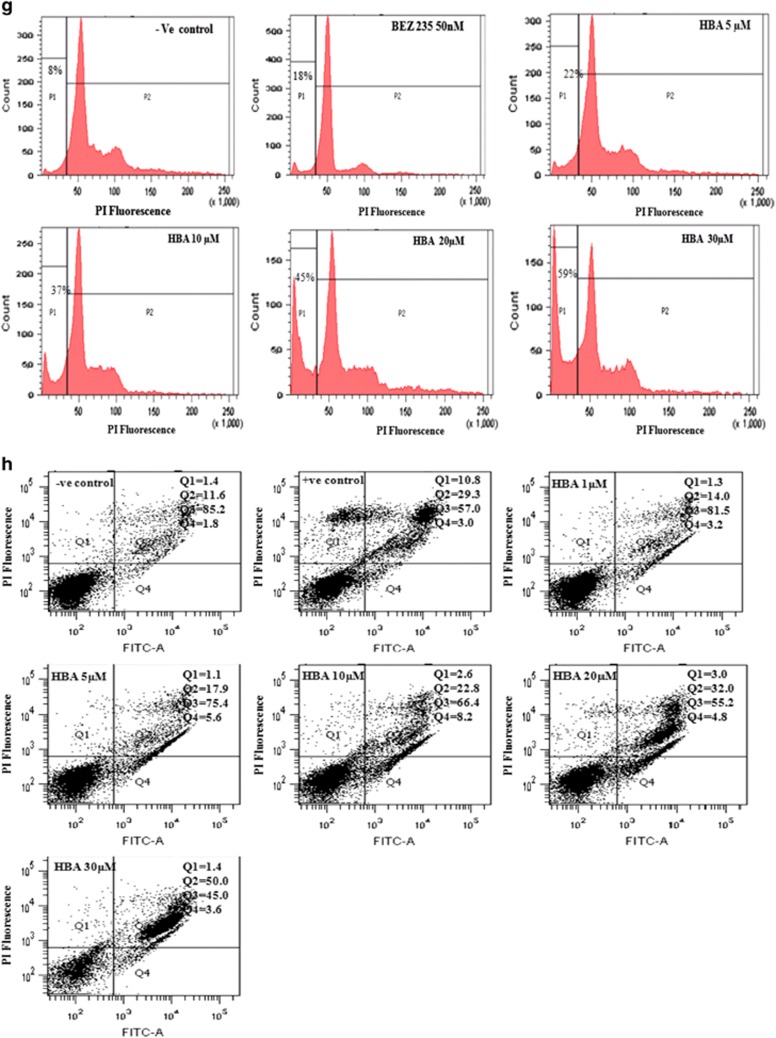

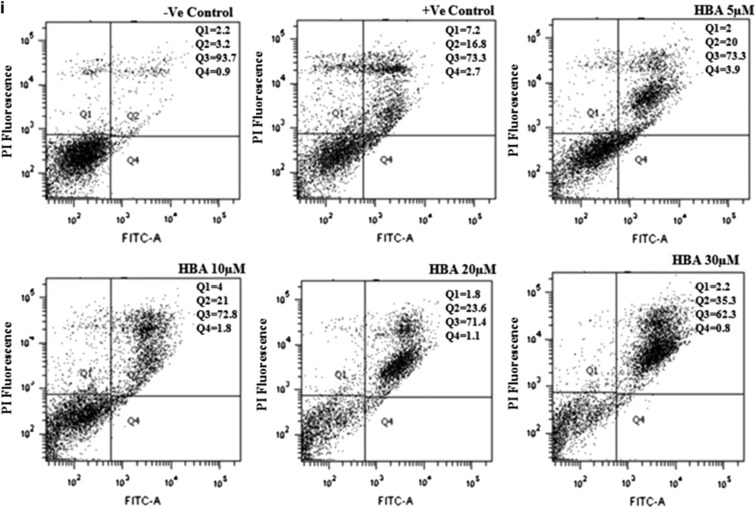
(**a**–**c**) Influence of HBA on the expression of proteins involved in cell cycle regulation. HL-60, THP-1 and MCF-7 cells were treated with the indicated concentrations of HBA for 24 h. *β*-actin was used as an internal control to represent the same amount of proteins applied for SDS-PAGE. Specific antibodies were used for the detection of cyclins A, E and D levels. Data are representative of one of two similar experiments. ***P<0.001, **P<0.01, *P<0.05 *versus* control using Student's *t*-test. (**d**) Confocal immunofluorescence done on MCF-7 cells. Cells were treated with HBA and after completion of the treatment cells were incubated with primary and secondary antibodies. Nuclear staining was done with DAPI. (**e**–**g**) Effect of HBA on cell cycle phase distribution. HL-60, THP-1 and MCF-7 cells in culture were treated with the indicated concentrations of HBA for 24 h. Cells were stained with PI to determine DNA fluorescence and cell cycle phase distribution by flow cytometery as described in Materials and Methods. Fraction of cells for hypo diploid (sub-G0, ≤2n DNA) population indicative of DNA damage was analyzed from PI *versus* cell counts shown (%). Data are representative of one of two similar experiments. (**h** and **i**) Estimation of early and late apoptotic cell population using annexin V–FITC staining. HBA-treated HL-60, THP-1 and MCF-7 cells was analyzed using annexinV–FITC staining. Cells were incubated with the indicated concentrations of HBA for 24 h and stained with annexin V–FITC and PI to analyze apoptotic and necrotic cell populations as described in Materials and Methods. Data are representative of one of two similar experiments

GSK3*β* is a substrate of PI3K/AKT pathway that is constitutively active in unstimulated cells.^13^ Keeping in view the importance of GSK3*β* in regulation of cyclins, CDKis and thus on the cell cycle, we sought to examine the phosphorylated levels of GSK3*β*. We have found significant inhibition of pGSK3*β* in HBA-treated HL-60 cells in a dose-dependent manner (Figure 4a). In THP-1 cells, the inhibition in the pGSK3*β* expression was observed at slightly higher concentrations. Under similar conditions, THP-1 cells treated with BA could produce only marginal effect at higher concentration of 30 *μ*M (Figure 4b). Looking into the expression profile of pGSK3*β* in MCF-7 cells, we found that HBA could inhibit this protein in a much similar manner to that observed in THP-1 cells (Figure 4c). The pGSK3*β* expression was also confirmed using fluorescence microscopy in MCF-7 breast cancer cells. The results clearly showed that in untreated cells pGSK3*β* is highly expressed in the cytoplasm after analyzing with nuclear counter stain DAPI (Figure 4d). Interestingly, HBA treatment induced a gradual decrease in the expression of pGSK3*β*. This inhibition of pGSK3*β* in response to HBA treatment coincides with its decreased phosphorylation that was observed through western blotting studies. As we have found inhibition in the expression of cyclins by treatment with HBA in all the three cancer cell lines, we wanted to study whether the treatment of HBA will affect the expression of any of the CDKis, and we observed that HBA treatment to the cancer cells of different histogenic origin caused drastic upregulation in the expression of p21 and p27 cip/kip proteins in a concentration-dependent manner (Figures 4a–c). BA the parent molecule also caused significant p21 and p27 protein upregulation at 30 *μ*M concentration in THP-1 cells (Figure 4b). Thus inhibition in the expression of cyclins was caused due to the upregulation in the expression p21/p27 cip/kip proteins that occurred due to inhibition in the phosphorylation of pAKT and thus inhibition in the downstream effector pathways. These observations provide strong evidence that inhibition of PI3K pathway by HBA in leukemia and breast cancer cells involve regulation of cell cycle by regulating cyclins and CDKis that induce cancer cell death.

### HBA induces cell cycle arrest and apoptosis in HL-60 cells, THP-1 and MCF-7 cells

Treatment of HL-60, THP-1and MCF-7 with HBA induced alterations in cell cycle phase distribution and DNA content. We observed changes in cell cycle distribution after 24 h treatment with various concentrations of HBA. The sub-G_0_ fraction was <6% in untreated control cells, which increased upto 57.7, 40.5 and 59% after treatment of the cells with 30 *μ*M concentration of HBA in HL-60, THP-1 and MCF-7 cells, respectively (Figures 4e–g). In THP-1 cells at 5 *μ*M concentration, we found 8.3% increase in the population of G_0_/G_1_ cells. This suggested that HBA might inhibit cell proliferation by inducing cell G_0_/G_1_ arrest, followed by apoptosis in THP-1 cells as was also confirmed by protein expression studies of cyclins. Apoptosis in live cells was detected by several other groups using different methods.^26, 27, 28^ For confirmation of apoptosis in live cells in our study, we performed annexin V–FITC staining in HBA-treated HL-60 cells and MCF-7. HL-60 and MCF-7 cells were incubated with different concentration of HBA for 24 h, and then cells were stained with annexin V–FITC and PI to assess the apoptotic and necrotic cell populations. As depicted in Figures 4h and I, compared with the untreated control early and late apoptotic cell populations has shown concentration-dependent increase upto 3.6 and 50% in HL-60 cells and upto 0.8 and 35.3% in MCF-7 at 30 *μ*M concentration of HBA, respectively, whereas in untreated control cells the early and late apoptotic cell populations was only 1.8 and 11.6% in HL-60 cells and 0.9 and 3.2% in MCF-7 cells.

### Modulation in Bcl-2/ Bax expression, generation of reactive oxygen species (ROS) and subsequent mitochondrial membrane potential (Δ*Ψ*_m_) loss in HL-60, THP-1 and MCF-7 cells

**Figure 5:**
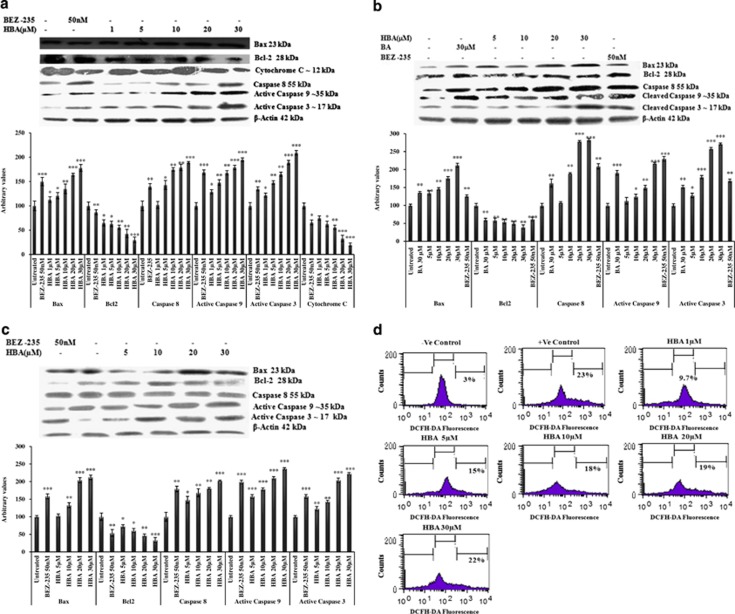

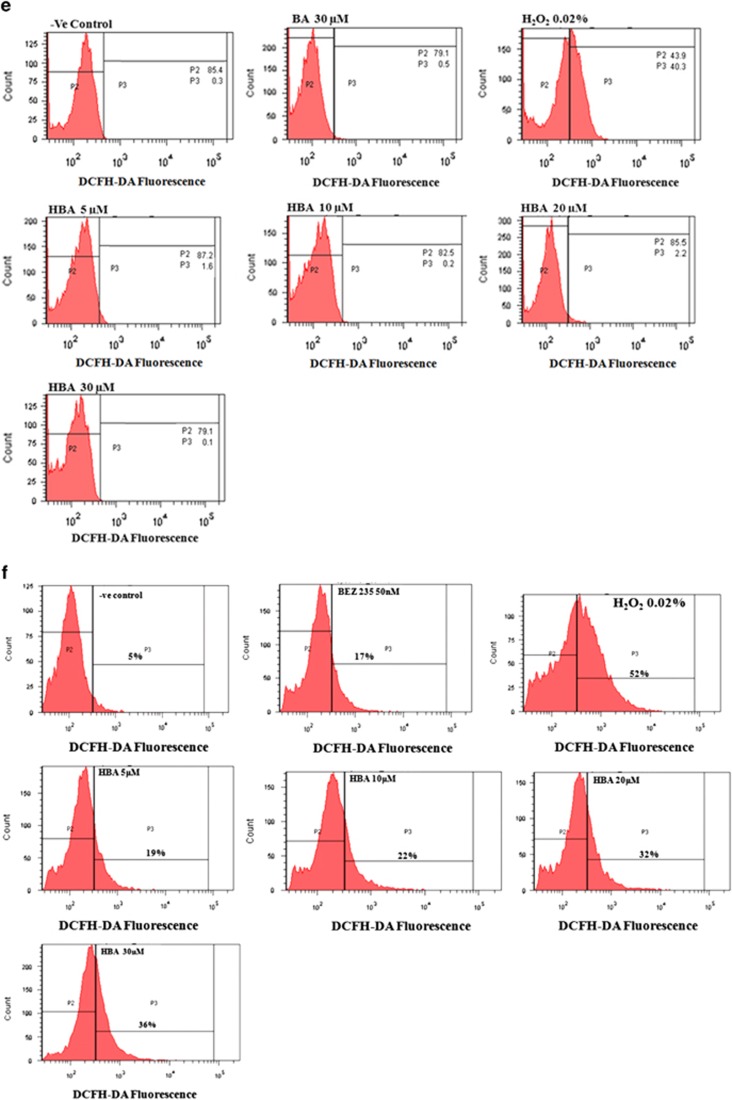

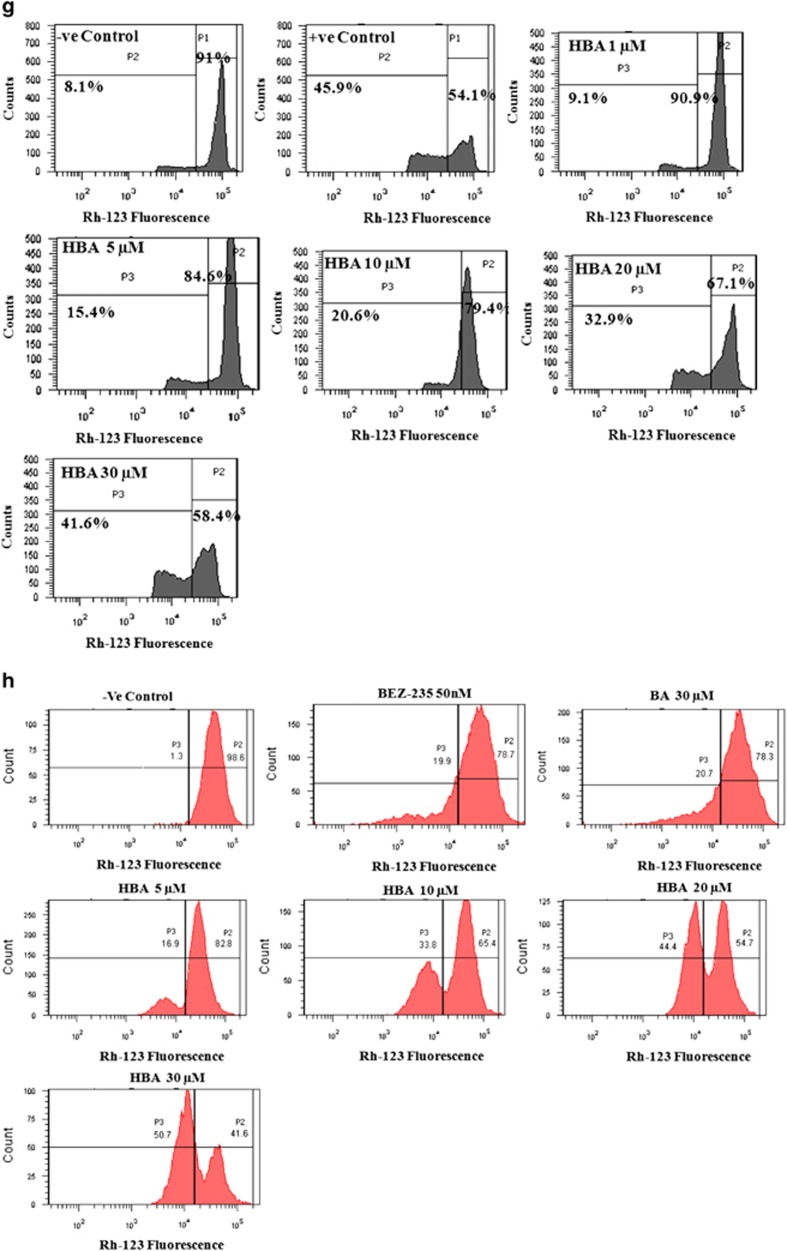

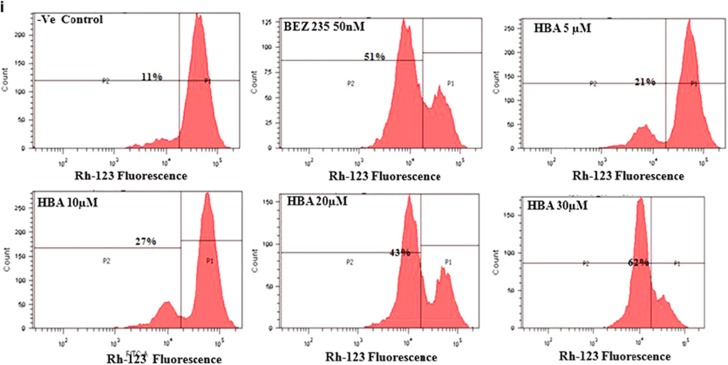
(**a**–**c**) Influence of HBA on the expression of proapoptotic and antiapoptotic proteins. HL-60, THP-1 and MCF-7 cells were treated with HBA, and the whole-cell lysate were prepared as described in Materials and Methods to observe the bcl-2/bax and cytochrome *c* expression. HBA induced activation of caspases in HL-60 cells, THP-1 and MCF-7 cells. Whole-cell lysate were prepared using HL-60 cells (2 × 106) treated with different concentrations of HBA for the indicated time periods, and 100 *μ*g/ml of protein were subjected to immunoblotting. Specific caspases 8 and 9 and caspase 3 antibodies was used for detection. Reprobing of the same membrane with the antibody against *β*-actin did not showed any change in the expression of this protein. Data are representative of one of two similar experiments. (**d**–**f**) HBA mediated early generation of ROS in HL-60, THP-1 and MCF-7 cells. Cells treated with the indicated concentrations of HBA for 24 h, followed by incubation with DCFH-DA (5 *μ*M) for 30 min. Cells were analyzed for DCF-fluorescence on flow cytometer in the FITC (DCF-HAD Fluorescence) *versus* count channels. Data are representative of one of two similar experiments. (**b**, **g**–**i**) HBA induced mitochondrial membrane potential loss (Δ*Ψ*m) in HL-60, THP-1 and MCF-7 cells. Cells were incubated with HBA at different concentrations in 6-well plate for 24 h. Cells were stained with Rodamine-123 (2 *μ*M) and analyzed on flow cytometer in the FITC (Rh-123 fluorescence) *versus* count channels. Data are representative of one of two similar experiments

A variety of physiological death signals as well as pathological cellular insults trigger the genetically programmed pathway of apoptosis.^29^ Moreover, PI3K pathway also leads to modulation in the activity of bcl2 family proteins; here we investigated the downstream antiproliferative and the proapoptotic effects of HBA in HL-60, THP-1 and MCF-7 cells by looking into the expression profile of bcl2 family proteins. Proapoptotic protein bax was upregulated after incubation with HBA, whereas the expression levels of antiapoptotic protein bcl2 was downregulated in all the three cancer cell lines (Figures 5a–c). In the case of THP-1, the expression of bcl2 family proteins got significantly modulated even at 5 *μ*M concentration, and the same expression profile was observed in the case of MCF-7 cells treated with HBA. Moreover, to look into the effect of HBA in live cells, MMP loss and ROS generation studies were carried out in HL-60, THP-1 and MCF-7 cells. Treatment of HL-60, THP-1 and MCF-7 cells with HBA caused a disruption of the mitochondrial membrane potential and hyperproduction of ROS as was observed in live HL-60 and MCF-7 cells; however, no ROS production could be observed in THP-1 cells. HBA could lead to mitochondrial membrane permeabilization, which was confirmed by decrease in the cytochrome *c* expression in the mitochondrial fraction of HL-60 cells (Figure 5a). The flow cytometric analysis revealed that HL-60 cells exposed to different concentrations of HBA for 24 h caused increase in 2',7'-dichlorfluorescein-diacetate (DCFH-DA) fluorescence from 3% in untreated control to 22% at 30 *μ*M in HL-60 cells; however, in MCF-7 the DCFH-DA fluorescence in untreated control was 5% that increased upto 36% at 30 *μ*M concentration of HBA (Figures 5d and f). In addition, THP-1 cells could not generate ROS, and 0.02% H_2_O_2_ was used as a positive control in the experimental setup that showed upto 40% ROS generation and the same was upto 52% in MCF-7 cells (Figure 5e). Similar to HBA in THP-1 cells treated with 30 *μ*M concentration of BA, no ROS generation was observed. Further, rhodamine-123 (Rh-123) retention in mitochondria is driven by Δ*Ψ*_m_ that allows the determination of cell population with active integrated mitochondrial functions. In the untreated control cells, almost all cells were bioenergetically active as evidenced by high Rh-123 fluorescence. However, incubation of HL-60, THP-1 and MCF-7 cells with 30 *μ*M concentration of HBA caused decrease of Δ*Ψ*_m_ upto 41.6, 50.7 and 62%, respectively (Figures 5g–i). The loss of mitochondrial potential reflected a direct effect of HBA on mitochondrial function, suggesting that mitochondrial events may be involved in activation of caspases and the simultaneous cell death in HL-60, THP-1 and MCF-7 cells.

### Activation of caspases and PARP cleavage in HBA-treated HL-60, THP-1 and MCF-7 cells

**Figure 6:**
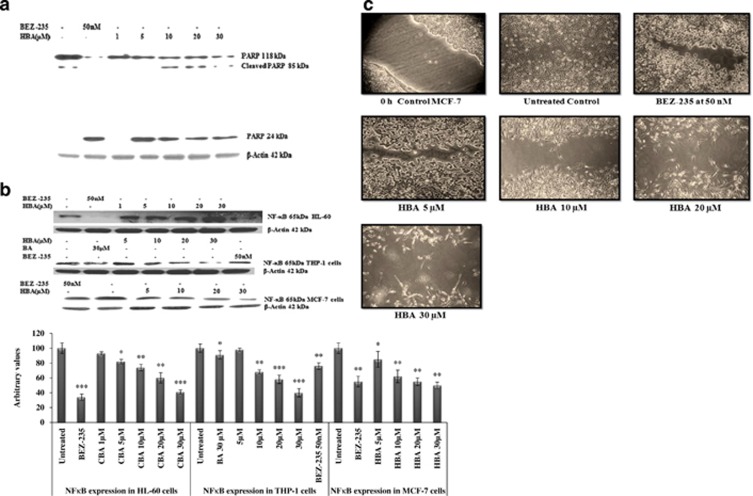
(**a**) Influence of HBA on the extent of PARP cleavage and apoptosis. Whole-cell lysate were prepared using HL-60 cells (2 × 10^6^) treated with different concentrations of HBA for the indicated time periods, and 100 *μ*g/ml of protein were subjected to immunoblotting. (**b**) Influence of HBA on the expression of transcription factor NF-*κ*B. Whole-cell lysate were prepared using HL-60 cells, THP-1 and MCF-7 cells (2 × 10^6^) treated with different concentrations of HBA for the indicated time periods, and 100 *μ*g/ml of protein were subjected to immunoblotting. Specific NF*κ*B antibody was used for the detection of NF*κ*B. Reprobing of the some membrane with the antibody against *β*-actin did not show any change in the expression of this protein. Data are representative of one of two similar experiments. ***P<0.001, **P<0.01, *P<0.05 *versus* control using the Student's *t*-test. (**c**) *In vitro* cell migration assay on MCF-7 cells. Briefly cells were seeded in six-well plate. When a tight cell monolayer was formed, a wound was created using a 1000-*μ*l tip, and the cell was then washed three times with PBS to remove cell debris. Cells were then treated with the indicated concentrations of HBA. After the completion of the treatment, fresh medium was added to the cells. The number of cells that migrated toward the wound was visualized using × 10 objective Olympus

Caspases are the members of the cysteine proteases, which are implicated in apoptosis and cytokine processing. HBA caused a strong increase in caspase activity, which peaked at 30 *μ*M concentration. We observed significant upregulation of active caspase 8 in HL-60, and the similar pattern was observed in THP-1 cells; however, in MCF-7 cells the upregulation pattern was not that significant as was observed in HL-60 and THP-1 cells (Figures 5a–c). In the case of MCF-7 cells, the active caspase 9 upregulation was observed even at 5 *μ*M concentration; however, in HL-60 and THP-1 the similar pattern was observer at higher concentrations. We also found significant active caspase 3 upregulation in all the three cancer cell lines. Comparing the upregulation of all the three caspases by BA and HBA, HBA could upregulate the caspase activity several fold higher than BA as was observed in THP-1 cells. PARP-1 is specifically proteolysed by caspases to a 24-kDa DNA-binding domain and to an 85-kDa catalytic fragment during the execution of the apoptotic program, which essentially inactivates the enzyme by destroying its ability to respond to DNA strand break. In order to further validate the extent of apoptosis induced by HBA; we wanted to see whether PARP has cleaved into its fragments. HL-60 cells were exposed to different concentrations of HBA for 24 h. HBA induced PARP cleavage into 85 and 24 kDa at 5 *μ*M concentrations that became more prominent at the 20 and 30 *μ*M concentrations (Figure 6a). BEZ-235 used as the positive control induced significant PARP cleavage when used at 50 nM concentration for 24 h.

### Inhibition of the NF*κ*B expression

NF*κ*B is a transcription factor that can be induced by a variety of signals. Understanding the significant involvement of NF*κ*B in maintaining the cell survival, we have tried to monitor the effect of HBA on the expression profile of this protein in HL-60, THP-1 and MCF-7 cells. Treatment of HL-60 cells with HBA significantly inhibited the expression of transcriptional factor NF*κ*B and the highest significant inhibition was observed at 30 *μ*M concentration. However, in the case of THP-1 and MCF-7, NF*κ*B transcription factor was inhibited even at 10 *μ*M concentration of HBA comparing with the parent BA, which was unable to show comparable inhibition at 30 *μ*M concentration (Figure 6b). BEZ-235 used as the positive control also showed significant inhibition of NF*κ*B protein expression in all the three cancer cell lines at a concentration of 50 nM for 24 h.

### Cell migration assay

Tumor invasion and metastasis require, among other cell behaviors, enhanced cancer cell mobility.^30^ In order to assess the effect of the compound on the migratory properties of cancer cells, MCF-7 cell monolayers were wounded by scratching and treated with HBA for 24 h. Cell cultures were photographed, and cell migration was assessed by comparing the gap sizes between the control and the treated wells. In the HBA-treated MCF-7 cells, the number of invasive cells that penetrated the respective wound was inhibited, and cell migration got significantly stopped. However, at higher concentration the cell morphology get altered due to the inhibitory effect of the compound HBA on cell migration and invasion (Figure 6c). Similar effects has also been found in MCF-7 cells. Thus our study has implications for rationale approaches to limiting cancer invasion and metastasis.

### Pharmacodynamics and estimation of acute toxicity by BA and HBA

Toxicological studies help to make an important decision about the use of new chemical entity as clinically effective and safe drug. Sub-acute oral toxicity has been advocated as a fundamental test for assessing safety, which has been most often applied previously in many safety assessment studies.^31^ In the development of BA derivative as an antineoplastic agent, we were keen to observe whether BA or HBA has any toxicity associated with them. It was interesting to observe that both BA and HBA did not show any treatment-related toxic manifestations and mortality up to the dose of 2000 mg/kg as compared with the vehicle control animals. The animals did not show any changes in the general appearance during the observation period. Also no significant change was observed in weekly body weight, feed (Table 1a), water consumption and any of the biochemical (Table 1b) or hematological parameters (Table 1c).

### Effect of HBA and BA on Ehrlich solid tumor

*In vivo* anti-tumor activity of HBA and BA against EAC (Ehlirch ascetic carcinoma) was evaluated by injecting 10 × 10^6^ EAC cells intramuscularly in the right thigh of Swiss male mice. As shown in Table 2, on thirteenth day, the tumor growth inhibition was calculated after treatment with HBA and BA on the basis of average tumor weight at a dose of 50 mg/kg i.p. and 100 mg/kg i.p. for BA and 40 mg/kg i.p. for HBA. For BA, the tumor-growth inhibition was found to be upto 39.00% and 28.05% at a dose of 100 mg/kg i.p. and 50 mg/kg i.p., respectively, as 1031.5±54.21 and 1215±51.8 mg average tumor weights were measured as compared with 1689.95±88.86 mg of the control group. For HBA, the tumor-growth inhibition was found to be upto 33.96% at a dose of 40 mg/kg i.p., and an average tumor weight was found to be 1116±96.78 mg as compared with 1689.95±88.86 mg of the control group. From the results, it can be inferred that compared with BA showing 28.05% tumor-growth inhibition at a dose of 50 mg/kg i.p. HBA is capable of showing higher tumor-growth inhibition of 33.96% at even lesser dose of 40 mg/kg i.p. This stresses upon the more potent anticancer potency of HBA molecule relative to BA in EAT model. Under similar conditions, 5-FU, which was used as a positive control, showed 52.05% tumor regression at a dose of 22 mg/kg i.p. Comparisons were made between the control and treated groups using the Student's *t*-test.

## Discussion

Cancer is the most deadly disease or, to be more appropriate, a group of diseases responsible for the majority of human deaths all over the globe.^32,33^ As the existing chemotherapeutics still possess some shortcomings such as the off-target effects.^34^ So, the need of the current scenario of cancer drug development is to develop lead molecules with selective inhibition of cancer cell growth. With the understanding of role of natural products and natural product modification in the present cancer drug discovery, we have synthesized a novel semi-synthetic triazoline derivative of BA (HBA).^20^ In the light of promising therapeutic potential of HBA reported by us, we were enthusiastic to explore the novel insight in deciphering the mechanisms involved in HBA-induced cell cycle arrest and apoptosis culminating in cancer cell growth inhibition. It was observed using different screening methods that HBA exhibited a greater antiproliferative and cytotoxic activity in a concentration- and time-dependent manner that was also confirmed by the inhibition of colony-forming ability of MCF-7 cells by HBA in the present study.

Numerous proteins have been implicated as having a crucial role in metastatic cancers. The present study identified a novel molecule with the ability of selectively killing the cancer cells via the inhibition of the PI3K/AKT signaling pathway in HL-60 cells, THP-1 and MCF-7 that represents an important anticancer target.^35^ In addition, as different PI3K isoforms have markedly different roles in cellular signaling, growth and oncogenic transformation, therefore ablation of a particular isoform will have its own downstream signaling effects.^36^ In the present case, HBA significantly downregulated the expression of both PI3K p110*α* and p85*α* in HL-60, THP-1 and MCF-7 cancer cell lines when compared with the untreated control. When comparing the effects of the parent molecule BA on the expression of PI3KP110*α* and P85*α*, we found that comparable effects were produced by BA at 2–3-fold higher concentration than produced by HBA at 20 and 30 *μ*M concentrations. Further moving downstream of PI3K/AKT signaling pathway, PI3K in its active form generates phosphotidyl inositol triphosphate (PI(3,4,5)P3. PI(3,4,5)P3 brings two PH domain-containing serine/threonine kinases, phosphoinositide-dependent kinase 1 (PDK1) and AKT into close proximity, phosphorylating AKT and thus activating it.^37^ AKT is the primary mediator of PI3K-initiated signaling and has a number of downstream substrates that may contribute to malignant transformation.^13^ HBA caused appreciable downregulation of pAKT in a dose-dependent manner in all the three cancer cell lines. Inhibition of pAKT induced by HBA on HL-60, THP-1 and MCF-7 was clearly evident that further explains why significant inhibition of PI3K/AKT increased apoptosis induced by HBA in these cancer cell lines.

As PI3K/AKT pathway is known to induce cell cycle progression.^13^ Cell cycle is regulated by highly multifaceted proteins that include cyclins, CDKs as well as CDKis.^38^ This prompted us to study the expression profile of cyclins in the present study and we could observe that HBA induced cell cycle arrest by downregulating the expression of cyclins D, E and A in HL-60, THP-1 and MCF-7 cells suggests that these proteins have been involved in cell cycle progression in leukemia and breast cancer cell lines employed. Overall, these results indicate that the decreased expression of cyclins contributes to the PI3K/AKT-mediated G_0_/G_1_ arrest. Importantly, HBA-induced cell cycle arrest is mediated by downregulation of cyclins, which may subsequently result in the decreased activity of CDKis. CDKis appears to be instrumental for maintaining human leukemia and breast cancer cells in the G_0_/G_1_ phase as was evident by the upregulation of p21/cip and p27/kip CDKi in HBA-treated cell lines in the present study. In order to further explore the mechanism of regulation of cell cycle by HBA, we looked into the expression status of pGSK3*β*. In our results, substantial downregulation of pGSK3*β* was observed in a dose-dependent manner keeping this protein in its active unphosphorylated state, suggesting that cell cycle arrest stimulated by HBA is induced by CDKi, which is encouraged by GSK3*β*. To further examine the effect of HBA on cell cycle in leukemia and breast cancer cells, flow cytometry analyses were performed, where results showed cells were arrested in the G_0_/G_1_ phase of the cell cycle followed by a significant increase of THP-1, HL-60 and MCF-7 cells in the sub-G_0_/G_1_ phase.

The mitochondrial involvement has been validated by looking into the expression of mitochondrial bcl-2 family proteins. AKT can modulate bad activity by phosphorylating at Ser^112^,^39^ and phosphorylated Bad then dissociates from bcl2 to form a complex with the 14-3-3 adaptor protein leading to cell survival.^40^ We observed decrease in the expression of bcl2 protein in all the three cancer cell lines that was associated with elevated levels of bax protein in a dose-dependent manner. Moreover, the involvement of mitochondria was also confirmed by the observed decrease in the expression of mitochondrial cytochrome *c* in HL-60 cells. The modulation in the expression of bcl2 family proteins lead to generation of ROS, which induced disruption of mitochondrial function with concurrent loss of Δ*Ψ*_m_. In addition, HBA-induced activation of caspases 8, 9 and 3 in HL-60, THP-1 and MCF-7 cells suggested both mitochondrial-dependent and -independent factors responsible for its apoptotic potential; however, the mitochondrial involvement is more pronounced. Mitochondrial apoptosis is primarily regulated by molecular interactions between different members of the bcl-2 family. There exist a link between mitochondrial and receptor-mediated apoptosis, and this link is provided by active caspase-8. This was observed in the present study where activation of caspase 8 helped in the pronounced mitochondrial-mediated apoptotic cell death in HL-60, THP-1 and MCF-7 cells. Subsequently, the activation of caspase cascade leads to the formation of cleaved products of PARP. Furthermore, NF*κ*B is a transcription factor that can be induced by a variety of signals.^41^ NF*κ*B expression status has been checked in HBA-treated HL-60, THP-1 and MCF-7 cells, and we could find appreciable decrease in the expression of NF*κ*B transcriptional factor in the present study. Our results demonstrate that treatment of HL-60 cells with HBA caused apoptotic cell death as was evidenced by DNA fragmentation in a concentration-dependent manner. Moreover, the induction of apoptosis was specific to leukemia cells only as no such laddering pattern had been observed in CV-1 cells, which is a normal monkey kidney cell line.^20^

Keeping the importance of the molecule into consideration, the acute toxicity studies were carried out. The study was designed and aimed to evaluate whether exposure of HBA is safe and not altering the activities of major physiological systems in comparison to BA, with the intention that the results would provide information on the safety of the compound. It has been observed that both BA and HBA were toxicologically safe as both of them did not cause any mortality or any adverse effect during testing. Furthermore, the biochemical parameters studied do not show any significant deviation from the control values. Therefore the present findings suggest that both HBA and BA are non-toxic compounds upto the dose of 2000 mg/kg body weight that correlates with the observation obtained under *in vitro* conditions where no apoptosis has been observed in normal monkey kidney (CV-1) cells treated with HBA.^20^ Furthermore, *in vitro* studies paralleled with the *in vivo* testing of HBA and BA against murine tumor models Ehrlich tumor (solid). The tumor-growth inhibition observed after treatment with HBA and BA showed substantial decrease. Comparing the *in vivo* anti-tumor potential of HBA with BA, we could find that HBA showed comparatively higher tumor-growth inhibition (33.96%) than BA (28.05%) when used at even lesser dose 40 mg/kg i.p. than BA (50 mg/kg i.p.). Therefore *in vitro* and *in vivo* studies highlighted the usefulness of HBA in treating human leukemia and breast cancers specifically by targeting the PI3K/AKT pathway.

In conclusion, our study tried to unveil a therapeutic potential of HBA, a triazoline derivative of BA in the tumor biology. We showed that the robust inhibition of the PI3K/AKT pathway can inhibit tumor progression by regulating the expression of cell cycle regulatory proteins and promote cell death in apoptosis-resistant cancers and therefore raise the potential usefulness of HBA as an excellent anticancer therapeutic candidate.

## Materials and Methods

RPMI-1640 medium, Rh-123, propidium iodide (PI), 3-(4,5-dimethylthiazole-2-yl)-2,5-diphenyltetrazolium bromide (MTT), eukaryotic protease inhibitor cocktail, penicillin, streptomycin, camptothecin, fetal bovine serum, sodium bicarbonate, phosphate-buffered saline (PBS), Tris buffer saline with tween-20 (TBST), radio immune precipitation assay lysis buffer, trypsin, electrophoresis reagents, protein estimation kit and protein marker were purchased from Sigma Aldrich Co. (St. Louis, MO, USA). Hyper film is from Amersham Biosciences (Buckinghamshire, UK). DNase-free RNase is from USB Corporation (Cleveland, OH, USA). Tris buffer and bromophenol blue were procured from Himedia (Mumbai, India). Glacial acetic acid was purchased from Fisher Scientific (Waltham, MA, USA), and trichloroacetic acid from Merck Specialties Private Ltd. (New Delhi, India). Annexin V–FITC apoptosis detection kit, anti-pAKT, anti-tAKT, anti-cyclin D, anti-pGSK3*β*, goat anti-rabbit IgG HRP, rabbit-anti-goat IgG HRP, goat anti-mouse IgG HRP and goat anti-mouse IgG-TR were purchased from Santa Cruz Biotechnology (Dallas, TX, USA). Anti-NF-kB anti-PI3K p110*α* and p85*α*, anti-Bax, anti-Bcl2, anti-caspase 8, anti-p27, anti-p21, anti-PARP antibodies and ECL Plus western blotting detection kit were purchased from Millipore Life Science Research (Darmstadt, Germany). Anti-*β*-actin antibody were purchased from Sigma Aldrich Co.

### Cell culture, growth conditions and treatment

Human promyelocytic leukemia cell line (HL-60), human breast cancer cell line (MCF-7) and human acute monocytic leukemia cell line (THP-1) used in the study were purchased from European Collection of Cell Culture (ECACC, Salisbury, UK). Cells were grown in RPMI-1640/MEM/DMEM containing 10% FCS, 100 unit Penicillin/100 *μ*g Streptomycin per ml medium. Cells were grown in CO_2_ incubator (Thermo Scientific, Waltham, MA, USA) at 37 °C with 98% humidity and 5% CO_2_ gas environment. Cells were treated with BA and its different structural analogs dissolved in DMSO while the untreated control cultures received only the vehicle (DMSO, <0.2%).

### Cell proliferation assay

HL-60, THP-1 and MCF-7 cells were grown in 96-well plates at a density of 1.5 × 10^4^cells/well/100 *μ*l and exposed to 1, 5 10, 20 and 30 *μ*M concentration of HBA and 1, 10 20, 30 and 40 *μ*M concentration of parent BA molecule for the different time intervals thereafter, and 20 *μ*l of MTT solution (2.5 mg/ml) was added to each well and incubated at 37 °C for 3–4 h in a humidified atmosphere containing 5% CO_2_. The plates were then centrifuged at 1500 r.p.m. for 15 min, and the supernatant was discarded while the MTT-formazon crystals were dissolved in 150 *μ*l DMSO. The OD was measured at 570 nm, with reference wavelength of 620 nm.^20^

### *In vitro* clonogenic assay

Clonogenic assay was performed to evaluate the ability of a cell to grow into a colony. MCF-7 cells were harvested from logarithmically growing stock cultures and plated at the required final clonogenic dilutions prior to treatment. After 24 h, the cells were treated with the indicated concentrations of the compound for another 24 h. After treatment, the plates were placed in an incubator for a time equivalent to at least six potential cell divisions (to give colonies of >50 cells). After this, the medium was aspirated from the plates so as not to disturb the colonies. Plates are then washed with PBS, and then a mixture of 6% glutaraldehyde and 0.5% crystal violet were added for 30 min to allow sufficient staining. The mixture was removed after 30 min, and the plates were washed with water and allowed to dry at room temperature.^20^

### Preparation of whole-cell lysates for immunoblotting

Cells (20 × 10^5^/well/2 ml) were collected, washed with cold PBS and incubated with cold lysis buffer and 1% (v/v) eukaryotic protease inhibitor cocktail for 45 min on ice. Cells were centrifuged at 14 000 *g* for 15 min to pallet the cell debris. Supernatant were transferred to a new tube and used as whole-cell lysates for western blotting analysis of various proteins.

### Protein measurement

Protein was measured by employing Sigma QuantiPro BCA kit using bovine serum albumin as the standard as per the instruction provided in the manual.

### Western blotting analysis

The whole-cell protein lysates were subjected to SDS-PAGE. Protein aliquots (100 *μ*g) were resolved on SDS-PAGE at 100 V for 2–3 h at room temperature and then electrotransferred to PVDF membrane at 120 V for 1.5–2 h maintained at 4 °C. Non-specific binding was blocked by incubation with 5% non-fat milk in PBS for 1 h at room temperature. Blocking of the membrane for phosphorylated antibodies was done by using 5% bovine serum albumin in PBS for 1 h. Blots were probed with respective primary antibodies overnight and washed three times with Tris buffer saline with 2% Tween-20 each for 5 min.^42^ The blots were then incubated with horseradish peroxidase-conjugated mouse, rabbit or goat secondary antibodies for 1 h, and again same washing steps were repeated each for 30 min. Signals were detected using ECL plus chemiluminescence's kit on X-ray film.

### DNA content and cell cycle phase distribution

HL-60, THP-1 and MCF-7 cells were seeded in six-well plates at a concentration of (10 × 10^5^ cells/well/2 ml) and treated with the indicated concentrations of HBA. After 24 h, cells were collected, washed in PBS and fixed in 70% cold ethanol overnight. Cells were again washed with PBS and subjected to RNase digestion (400 *μ*g/ml), and finally, cells were incubated with PI (10 *μ*g/ml). Cells are then analyzed immediately on flow cytometer FACS Diva (Becton Dickinson, Franklin Lakes, NJ, USA). The fluorescence intensity of sub-G_0_/G_1_ cell fraction represents the apoptotic cell population.^43^

### Flow cytometric analysis of apoptosis and necrosis using annexin V/PI dual staining

HL-60, THP-1 and MCF-7 cells after treatments with different concentrations of HBA for 24 h were collected and washed twice with cold PBS and resuspened in 400 *μ*l 1 × binding buffer. Thereafter 2 *μ*l of annexin V–FITC antibody were added to the cell suspension, and the cells were incubated for 30 min at 37 °C. Ten microliters of PI was added immediately before flow cytometric analysis. PI was used to distinguish cells that have lost membrane integrity. The cells were scanned for fluorescence intensity in FITC and PI channels.^44^

### Flow cytometric measurement of intracellular peroxides (ROS) generation in HL-60 cells

Intracellular peroxides level was determined by using 2, 7-dichlorofluoresceindiacetate. An increase in green fluorescence intensity is used to quantify the generation of intracellular ROS. Cells (10 × 10^5^/2 ml/six-well plates) were incubated with different concentrations of HBA. Before 30 min of the termination of the experiment, cells were incubated with DCFH-DA (5 *μ*M). Cells were washed in PBS and centrifuged at 1500 r.p.m. for 5 min and suspended in PBS. The green DCF-fluorescence was analyzed in the FL-1 channel (excitation *λ* 488 nm; emission *λ* 535 nm).^44^

### Measurement of mitochondrial membrane potential for cellular energy status

Changes in Δ*Ψ*_m_ as a result of mitochondrial perturbation were measured with Rh-123 staining. HL-60, THP-1 and MCF-7 cells at a density of (10 × 10^5^/ml/well) were grown in six-well plates and treated with the indicated concentrations of HBA. Rh-123 (2 *μ*M) was added 30 min before the termination of experiment. Cells were washed in PBS and centrifuged at 1500 r.p.m. for 5 min and suspended in PBS. The decrease in intensity of fluorescence from 10 000 events because of mitochondrial membrane potential loss was analyzed in FITC channel on flow cytometer.^45^

### *In vitro* cell migration assay

The wound-healing assay is simple, inexpensive and one of the earliest developed methods to study directional cell migration *in vitro*. The basic steps involve creating a ‘wound' in a cell monolayer, capturing the images at the beginning and at regular intervals during cell migration to close the wound and comparing the images to quantify the migration rate of the cells. In all, 30 × 10^4^cells/ml/well were seeded in six-well plates. Cells were then wounded by scratching with a 1000-*μ*l pipette tip and the cell was washed three times with PBS to remove cell debris. HBA was added at the desired concentration, and the plates were incubated in the CO_2_ incubator for another 24 h. After the completion of the treatment, fresh medium was added into the plates, and the plates were again incubated till the control wound gets completely healed. The wounds were photographed ( × 10 objective) at 0 and 24 h, and healing was quantified by measuring the distance between the edges of the wound.^26^

### Pharmacodynamics and acute toxicity measurements

BA and HBA were evaluated for oral acute toxicity as per the rules of the Organization for Economic Cooperation and Development guidelines in female Swiss mice.^46^ Briefly, five female swiss mice per group received BA and HBA starting at 2000 mg/kg via oral route. The animals were kept in perspex chambers and monitored for any toxic symptoms for the first 4 h and then intermittently up to 24 h as described previously.^47^ Animals were further observed daily for the next 13 days for mortality or observable toxicity, if any. Finally, the number of survivors was noted. Body weight changes were recorded weekly, and feed and water intake was measured on daily basis. At the end of the study, the serum was assayed for determination of various biochemical parameters such as glucose, alkaline phosphatase, urea, and so on using commercial kits (Erba, Manheim, Germany). Moreover, investigation of the whole blood was done for various hematological parameters such as red blood cells, white blood cells, hemoglobin, platelet count, hematocrit, mean corpuscular volume, mean corpuscular hemoglobin concentration, neutrophils, lymphocytes, monocytes, eoisinophils and basophils.^48^

### *In vivo* anti-tumor activity against Ehrlich tumor solid

*In vivo* anti-tumor activity of BA and HBA was evaluated in murine tumor models. Swiss albino mice, were maintained at 23 °C temperature with 20–25 complete air changes with 100% fresh air at 50–60% relative humidity. The number of animals used in the present study was approved by the Institutional Animal Ethics Committee, Indian Institute of Integrative Medicine, Jammu. EAC cells were collected from the peritoneal cavity of the Swiss mice harboring 8–10-days-old ascitic tumor. On day 0, 10 × 10^6^ EAC cells were injected intramuscularly in the Swiss albino mice (*n*=38) on the right thigh. The next day, animals were randomized and divided into five different groups. Four treatment groups contained 7 animals each and one control group contained 10 animals. The first test group was treated with 50 mg/kg, i.p. BA, second test group with 100 mg/kg i.p. BA and third group was received 40 mg/kg i.p. HBA. Fourth treatment group received 5-fluorouracil (22 mg/kg, i.p.), and it served as a positive control. The control group was similarly administered normal saline (0.2 ml, i.p.). On day 9 and 13, tumor-bearing thigh of each animal was shaved, and the longest and shortest diameter of the tumors were measured with the help of a vernier caliper. Tumor weight of each animal was calculated using the formula.


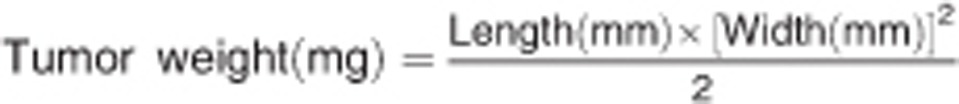


The percentage tumor growth inhibition was calculated on day 13 by comparing the average value of the treated groups with that of the control group. Tumor growth in saline-treated animals was taken to be 100%.

### Statistical analysis

Statistical analysis was done by using the Student's *t*-test, and *P*-value<0.05 was considered to be significant, with *P<0.05, **P<0.01 and ***P<0.001.

## Figures and Tables

**Table 1a:** Mean weekly feed and water consumed by female mice during the acute oral toxicity study of BA and HBA

**Treatment**	**First week**	**Second week**
*Weekly feed consumed (g)*		
Control	37.54±1.78	39.04±2.5
BA	39.14±5.3	43.98±7.04
HBA	38.58±2.85	42.88±3.87
		
*Weekly water consumed (ml)*		
Control	47.18±5.54	49.28±7.91
BA	46.66±7.04	50.65±8.84
HBA	46.7±5.51	50.05±9.43

Values are represented as mean±S.E.M. (*n*=7/group). Student–Newman–Keul's test and *T*-test (ANOVA)

**Table 1b:** Mean values of various biochemical parameters measured from serum of female mice treated with vehicle, BA and HBA (single oral dose)

**Parameters**	**Control**	**BA**	**HBA**
Glucose (mg/dl)	156.3±26.88	159.2±10.03	155.7±18.77
Triglyceride (mg/dl)	127.5±20.77	144±26.45	151.2±33.68
Cholesterol (mg/dl)	86.14±31.09	76.73±12.03	83.76±11.13
SGOT (IU/l)	141.45±7.22	147.93±19.31	121.085±24.21
Total Protein (g/dl)	4.67±0.91	6.83±0.48	5.16±0.08
Creatinine (mg/dl)	0. 53±0.05	0.57±0.06	0.483±0.09
Urea (mg/dl)	36.09±3.98	36.47±1.12	37.16±1.33
Bilirubin (mg/dl)	0.654±0.01	0.518±0.01	0.53±0.05
ALP (IU/l)	152.4±10.83	157.7±14.11	167.44±3.71

Values are represented as mean±S.E.M. (*n*=7/group). Student–Newman–Keul's test and *T*-test (ANOVA)

**Table 1c:** Mean values of various hematological parameters measured from whole blood of female mice treated with BA and HBA (single oral dose)

**Parameters**	**Control**	**BA**	**HBA**
WBC (10^3^/ul)	9.237±1.03	7.63±1.28	7.292±1.29
RBC (10^6^/ul)	7.97±0.14	9.258±0.3	9.124±0.26
HGB (g/dl)	11.67±0.26	12.8±0.45	13.06±0.33
HCT (%)	36.72±1.3	40.8±1.08	41.36±0.93
MCV (fl)	47.30±1.26	44.12±0.7	45.38±1.05
MCH (pq)	14.06±0.32	13.82±0.21	14.34±0.25
MCHC (g/dl)	29.72±0.45	31.36±0.33	31.6±0.29
PLT (10^3^/ul)	1409±149.7	1351±137.3	1433±34.3
NEUT (%)	13.8±2.03	14.96±2.32	16.96±2.65
LYMPH (%)	68.81±12.41	75.34±2.69	64.24±10.71
MONO (%)	5.8±2.22	6.48±1.02	5.22±0.65
EO (%)	1. 81±0.01	2.69±0.13	2.6±0.18
BASO (%)	0.1±0.02	0.1±0.04	0.008±0.02

Values are represented as mean±S.E.M. (*n*=7/group). Student–Newman–Keul's test and *T*-test (ANOVA)

**Table 2:** *In vivo* anti cancer activity of BA and HBA

	**Average body weights(g) of animals on days**			**Day 13**			
**Treatment groups**	**1**	**5**	**9**	**Av. body weights (g)**	**Av. tumor weights (mg)**	**% Tumor growth inhibition**	**Mortality**
BA (50 mg/kg i.p.)	20.14	21.71	21.81	22.57	1215.78±51.8**	28.05	0/7
BA (100 mg/kg i.p.)	20.28	21.28	22.00	22.28	1031.5±54.21***	39.00	0/7
HBA (40 mg/kg i.p.)	20.42	21.42	21.85	22.70	1116±96.78***	33.96	0/7
5-Florouracil-positive control (22 mg/kg i.p.)	20/71	21.42	20.71	20.85	817.5±33.71***	52.05	0/7
Normal saline NS (0.2 ml i.p.)	20.80	21.50	22.80	23.50	1689.95±88.68	—	0/10

Values are mean±S.E. (*n*=7, 10 for control). *P<0.05, **P<0.01, ***P<0.001 *versus* normal saline control

## Abbreviations

BA: betulinic acid
DCFH-DA: 2',7'-dichlorfluorescein-diacetate
HBA: *3{1*N*(5-hydroxy-naphth-1yl)-1*H*-1,2,3-triazol-4yl}methyloxy betulinic acid*
MTT: 3-(4,5-dimethylthiazole-2-yl)-2,5-diphenyltetrazolium bromide
NF*κ*B: nuclear factor kappa B
PI3K: phosphotidylinositol-3 kinase
PI: propidium iodide
ROS: reactive oxygen species
Rh-123: Rhodamine-123
